# A Rare Case of a Subgaleal Hematoma With Global Developmental Delay, Scurvy, and Congenital Toxoplasmosis, Rubella, Cytomegalovirus, Herpes Simplex, and HIV (TORCH) Infection

**DOI:** 10.7759/cureus.52211

**Published:** 2024-01-13

**Authors:** Ankita Patel, Jayant D Vagha, Revat J Meshram, Rahul Khandelwal

**Affiliations:** 1 Pediatrics, Jawaharlal Nehru Medical College, Wardha, IND

**Keywords:** interdisciplinary management, pediatric neurology, congenital torch infection, subgaleal hematoma, scurvy, global developmental delay

## Abstract

This case report details the complex presentation of a six-year-old female child with global developmental delay (GDD), scurvy, congenital toxoplasmosis, rubella, cytomegalovirus, herpes simplex, and HIV (TORCH) infection and a subgaleal hematoma. The patient's medical history included delayed developmental milestones, bilateral congenital cataract, and a previous generalized tonic-clonic seizure. Thorough investigations revealed cerebral atrophy, bilateral ventricular dilatation, and periosteal thinning consistent with scurvy. The interdisciplinary approach involving neurology, ophthalmology, and orthopedics resolved the subgaleal hematoma. This case underscores the intricate interplay of neurological, nutritional, and infectious factors in pediatric conditions and highlights the importance of a collaborative, multidisciplinary approach for accurate diagnosis and effective management.

## Introduction

Global developmental delay (GDD) encompasses a diverse range of conditions characterized by significant delays in achieving developmental milestones during early childhood. GDD may arise from various etiologies, such as genetic, neurological, metabolic, and environmental factors [1]. The complexity of GDD often necessitates a comprehensive diagnostic evaluation involving multiple medical specialties. Congenital infections, including toxoplasmosis, rubella, cytomegalovirus, and herpes simplex (TORCH), pose a substantial risk to fetal development during pregnancy. These infections, caused by *Toxoplasma gondii*, rubella virus, cytomegalovirus (CMV), and herpes simplex virus (HSV), are associated with a spectrum of congenital abnormalities, including neurological and developmental issues [2]. Early detection and timely intervention are imperative to mitigate the potential long-term consequences of TORCH infections.

Scurvy, a condition resulting from vitamin C deficiency, is commonly associated with impaired collagen synthesis, leading to musculoskeletal and vascular abnormalities. While scurvy is rare in the modern era, it can still occur in populations with inadequate nutrition or specific medical conditions affecting vitamin C metabolism [3]. Seizures in pediatric patients can have diverse etiologies, ranging from genetic factors to acquired conditions. Seizures in children with GDD may further complicate the clinical picture, warranting a comprehensive assessment to identify the underlying causes and guide appropriate management [4]. The prevalence of epilepsy is high in children with GDD, with 29.6% of patients in a study having GDD and epilepsy [4].

Subgaleal hematomas, though infrequently encountered, can occur following traumatic events, such as seizures. These hematomas are characterized by the accumulation of blood between the periosteum and the galea aponeurotica, often requiring prompt diagnosis and intervention to prevent complications [5]. The coexistence of GDD, congenital TORCH infection, scurvy, seizures, and subgaleal hematoma in a pediatric patient poses a diagnostic challenge. This case highlights the importance of considering a broad differential diagnosis and underscores the need for a collaborative, multidisciplinary approach to address the complexity of such cases.

## Case presentation

We present the case of a six-year-old female child who came to our attention with a complaint of swelling in the left parietal region for the past month. The child had a history of GDD, bilateral congenital cataract, and a previous generalized tonic-clonic seizure. She was born prematurely at 1.6 kg via normal vaginal delivery to a term primigravida. Despite the low birth weight, the patient cried immediately after birth. Although she did not require admission to the neonatal intensive care unit (NICU), she did stay in the hospital for an extended period due to her prematurity. The cause of premature birth and the details of the early hospital stay, including any supportive measures provided during that time, are important aspects of the patient's history that should be addressed. While the patient did not require NICU admission, her low birth weight suggests the need for clarification regarding her postnatal care. Notably, developmental concerns arose when the child failed to achieve milestones at six to seven months, prompting an evaluation by a neurologist. Subsequent investigations revealed bilateral congenital cataract and cerebral atrophy with a thinned corpus callosum on MRI.

According to the child's mother, the recent swelling in the left parietal region followed a seizure episode, during which the child hit her head on the floor. The mother reported bleeding gums and brought the child to the hospital due to the persistent swelling. Notably, the child's milestones were significantly delayed: she achieved independent sitting at 2.5 years, coordinated transfer at three years, bisyllabic language at four years, and exhibited social laughter at two years. The child had a history of a generalized tonic-clonic seizure one year prior.

**Figure 1:**
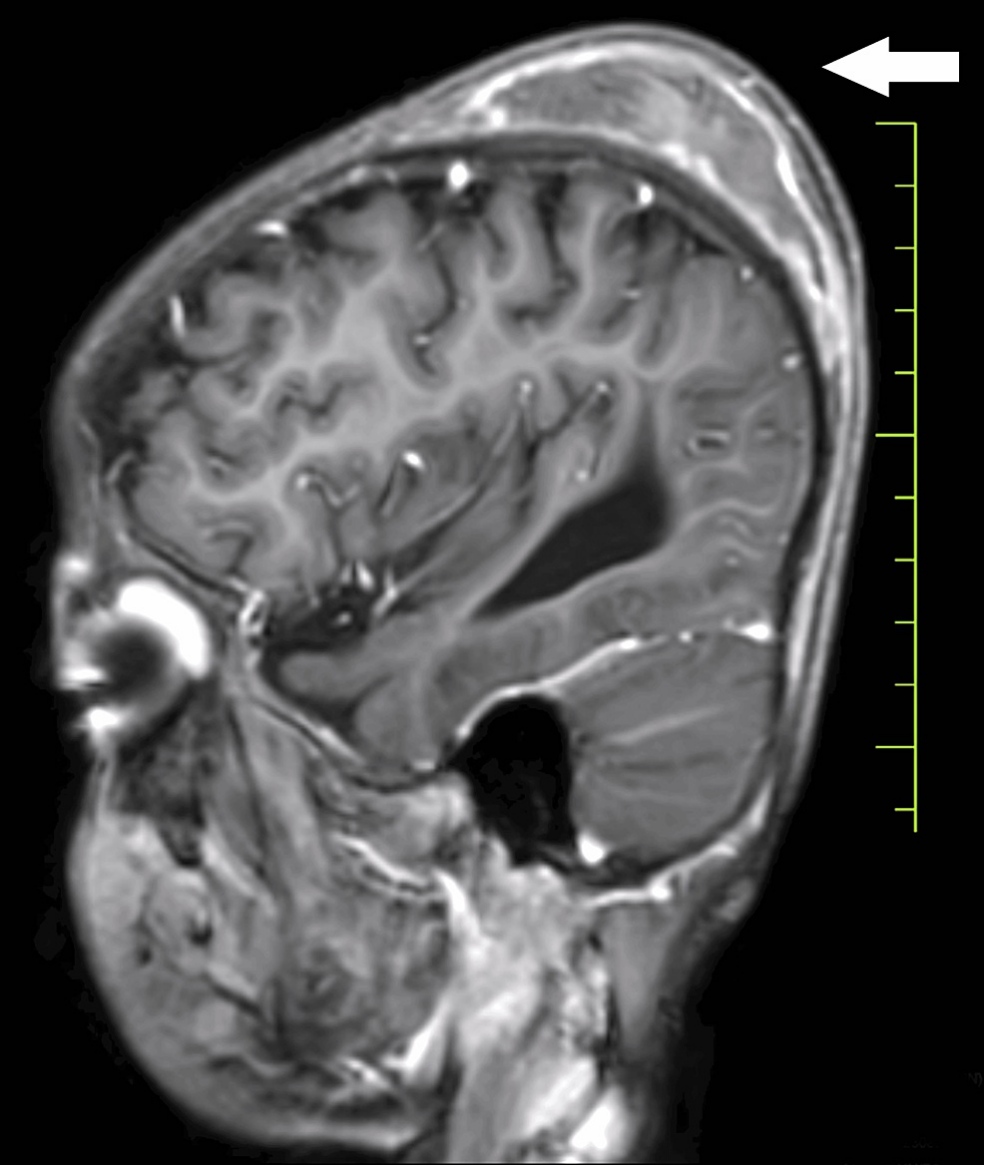
Subgaleal hematoma

**Figure 2:**
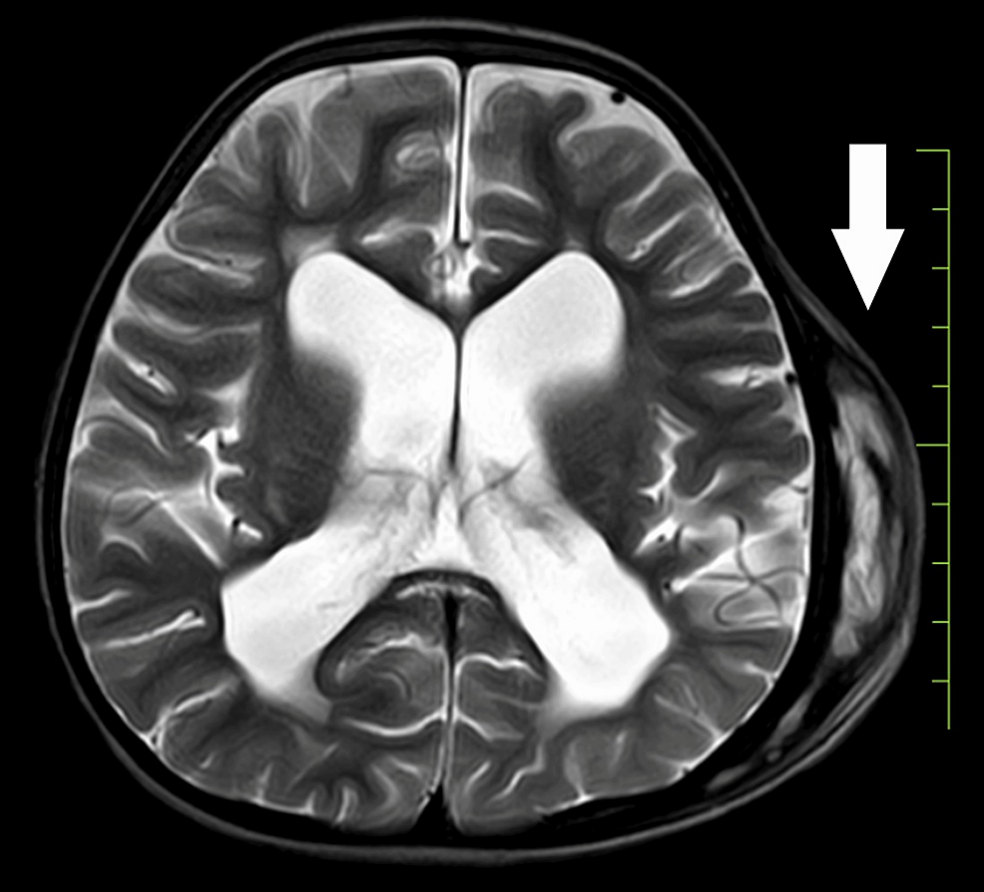
Bilateral ventricular dilatation

**Figure 3:**
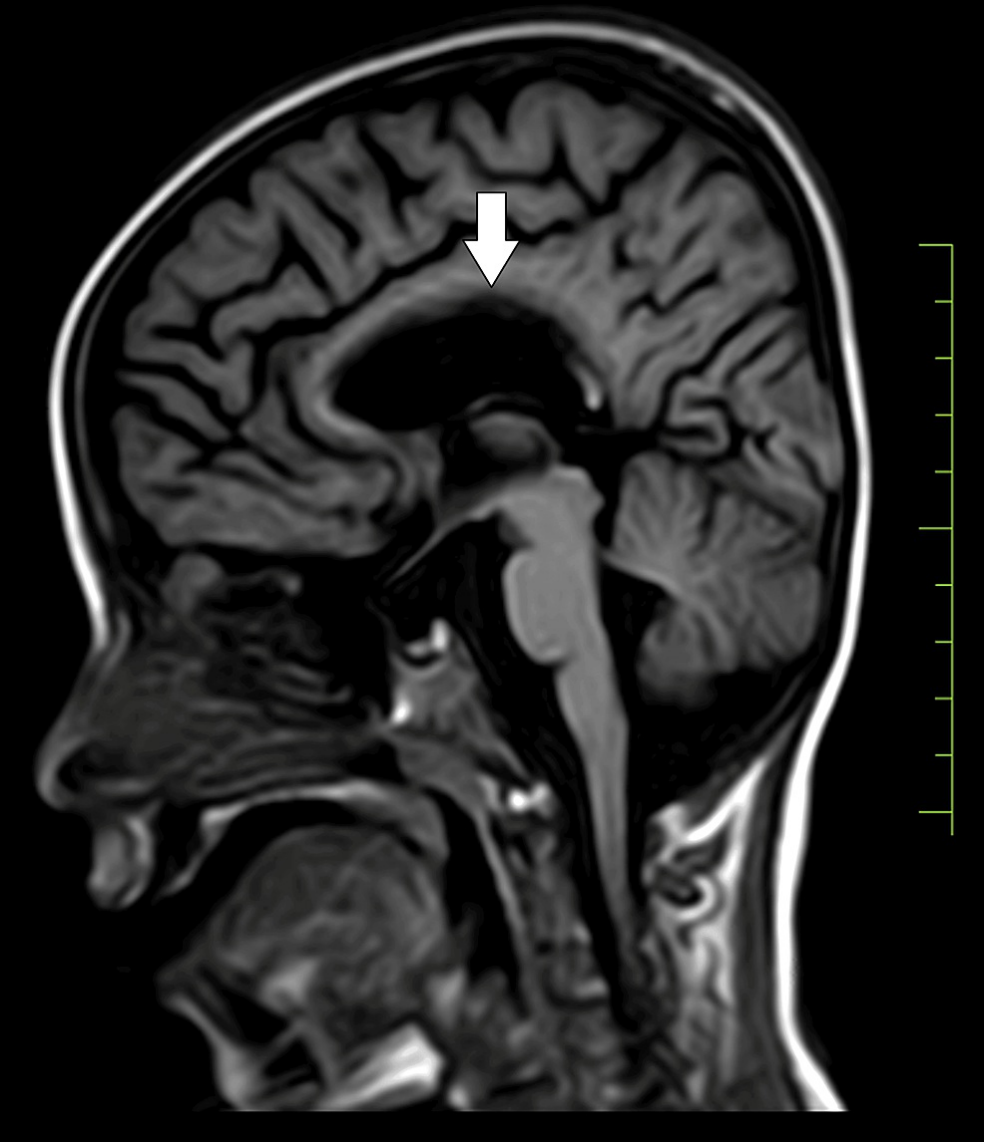
Thinned corpus callosum

**Figure 4:**
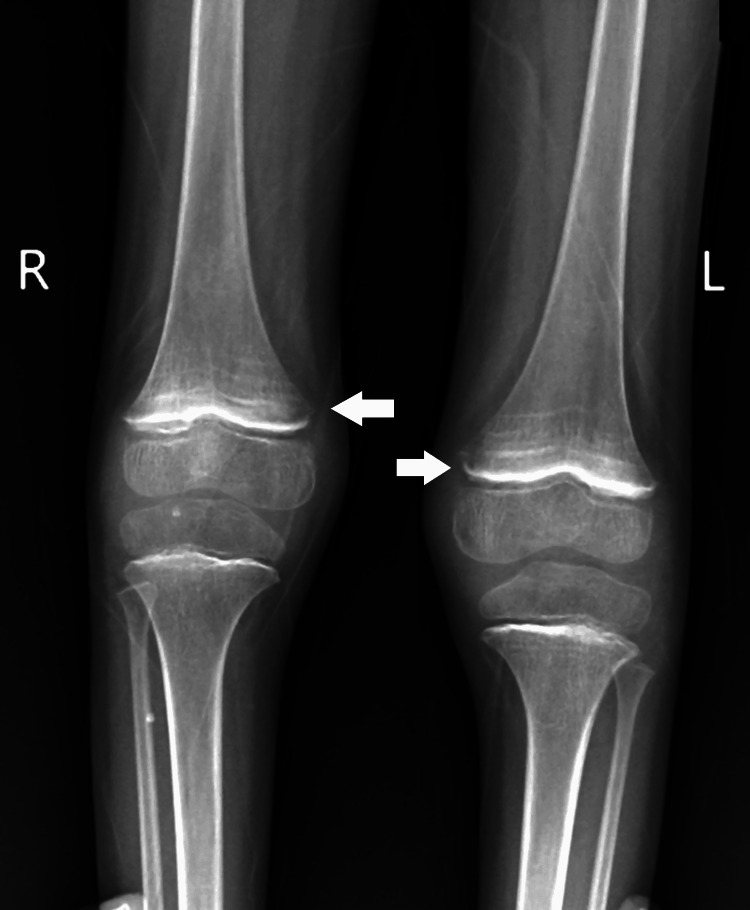
Periosteal thinning consistent with scurvy

Upon examination, the child displayed bilateral extensor plantar reflexes, bleeding gums, and tenderness in the legs suggestive of scurvy. Milestone assessment revealed delayed gross and fine motor skills, language development, and social interactions. Subsequent investigations, including a 2D echocardiogram (normal), MRI of the brain indicating subgaleal hematoma (Figure 1), bilateral ventricular dilatation (Figure 2), and thinned corpus callosum (Figure 3), and X-ray of the lower limbs showing periosteal thinning consistent with scurvy (Figure 4) collectively revealed a complex medical picture.

Following the multidisciplinary intervention, the patient exhibited significant improvement. Regular follow-up assessments indicated a resolution of the subgaleal hematoma. The child's seizure activity was effectively controlled with the injection of levetiracetam, and bleeding gums ceased with vitamin C supplementation (tab Limcee). In addition, the radiographic evidence of periosteal thinning in the lower limbs, indicative of scurvy, showed signs of recovery with a Syp combination of calcium, magnesium hydroxide, vitamin D3, and zinc sulphate. The child continued receiving ongoing developmental support to address GDDs, focusing on physical and cognitive therapies. Regular monitoring of milestones revealed progressive advancements, demonstrating the positive impact of the comprehensive treatment approach. This case emphasizes the importance of continuous follow-up and a collaborative, holistic approach in managing complex pediatric cases to achieve favorable outcomes.

## Discussion

The presented case underscores the intricate interplay of GDD, scurvy, and congenital TORCH infection in a pediatric patient with a subsequent subgaleal hematoma. The unique combination of these conditions necessitated a multidisciplinary approach for accurate diagnosis and effective management. Our patient's delayed achievement of developmental milestones aligns with the broader category of GDD. GDD is a heterogeneous condition encompassing various neurodevelopmental disorders, emphasizing the importance of thorough evaluation and collaboration among specialists [6]. The neurological findings, including cerebral atrophy and thinned corpus callosum on MRI, contribute to understanding the underlying neurological abnormalities associated with GDD [7].

The clinical presentation of bleeding gums, leg tenderness, and radiographic evidence of periosteal thinning in the lower limbs indicated coexisting scurvy. The correlation between scurvy and musculoskeletal abnormalities has been documented in pediatric literature [8]. Vitamin C deficiency, the cause of scurvy, can impair collagen synthesis, affecting connective tissues and bone development [9]. The positive TORCH panel results further complicated the case. TORCH infections during pregnancy can have profound consequences on the developing fetus, potentially leading to a spectrum of congenital abnormalities [10]. In this case, the congenital TORCH infection likely contributed to the patient's complex clinical presentation, necessitating a comprehensive diagnostic approach.

The occurrence of a subgaleal hematoma following a seizure episode adds another layer to the complexity of the case. While subgaleal hematomas are rare in pediatric populations, their association with traumatic events, including seizures, has been reported [11]. The need for prompt diagnosis and management is crucial to prevent potential complications. The successful resolution of the subgaleal hematoma and improvement in the patient's overall condition highlight the effectiveness of a multidisciplinary approach. Collaboration among neurology, ophthalmology, and orthopedics was pivotal in addressing the diverse clinical manifestations.

## Conclusions

This case illuminates a rare and intricate clinical scenario involving a six-year-old female child presenting with GDD, scurvy, and congenital TORCH infection, ultimately resulting in a subgaleal hematoma. The collaborative efforts across various medical specialties facilitated a comprehensive diagnostic evaluation and led to a successful multidisciplinary intervention. The patient exhibited significant improvement with the strategic use of antiepileptic medication, vitamin C supplementation, and calcium support. The resolution of the subgaleal hematoma and positive developmental trajectory underscore the efficacy of the chosen treatment strategy. Emphasizing the importance of recognizing the interconnectedness of diverse medical conditions in pediatric patients, this case prompts a deeper exploration into recent literature, shedding light on the association between scurvy and developmental disorders. This additional insight contributes to our understanding of the complex interplay between nutritional deficiencies and neurodevelopmental challenges in pediatric cases, reinforcing the ongoing need for meticulous follow-up and developmental support for optimal outcomes in such intricate scenarios.
